# Older adult supportive environment at home—a case study in Jordan: overall sense of control associated with home modification

**DOI:** 10.3389/fpubh.2024.1329315

**Published:** 2024-12-04

**Authors:** Majd Al-Homoud

**Affiliations:** ^1^Architecture Department, College of Architecture and Design, Prince Sultan University, Riyadh, Saudi Arabia; ^2^Department of Architecture, School of Architecture and Built Environment, German Jordan University, Amman, Jordan

**Keywords:** older adults, Jordan, housing market, cultural heritage, urban planning, sustainability, modification, urban policy

## Abstract

**Introduction:**

Modification becomes a critical issue in a supportive home environment for older adults.

**Methods:**

This study examined if modifications in different spaces at home are facilitated to provide a supportive environment for older adults to feel in control in a cultural context. Data was utilized using field research with a mixed method design: Structured and open-ended interviews as well as surveys using a questionnaire. The hypothesis states that older adults have a supportive environment at home when the overall sense of control is associated with home modification.

**Results:**

The result reveals that overall sense of control with home modification and its components: kitchen, bedroom, living room, and reception room have a significant effect on older adults’ wellbeing.

**Discussion:**

Therefore, older adult supported environment in Jordan can only take place by boosting awareness about the existing problems in the physical environment, by describing the benefits of home modification and by reducing the barriers of home modifications. This process can enhance the housing market and provide better urban policies for urban design and urban planning based on cultural heritage and values.

## 1 Introduction

Populations are expected to age rapidly in Arab countries, and age longevity is often accompanied by many years of ill health and disability. Yet most of the countries in the Arab region continue to rely on family members, especially women, as the primary source of older adult care (1). However, Arab countries are now experiencing demographic transitions including longer life expectancy. By 2050, the proportion of older persons (60 years or more) is predicted to increase to 19% compared to an average of around 7% in 2010. Because of the history of high fertility rates, the number of older persons is predicted to more than quadruple from 22 million in 2010 to 103 million by 2050 (2).

As people age, their needs increase, and the type and level of support may change also. As people age, they undergo several physiological changes, affecting how they look, function, and respond to daily living. Changes in the late-life span involve a general slowing down of all organ systems due to a gradual decline in cellular activity. Individuals experience these changes differently; for some, the level of decline may be rapid and dramatic, and for others, changes are much less significant (3).

Differentiation among cultures affects views towards older adults, their life expectancies, and care provision. Arab culture is influenced by Islamic values of how to care for older adults as they age. In the Arab culture, aging at home versus nursing homes is more acceptable, as older adults must age in their own homes. Although Arab homes are not fit to respond to older adults’ actual needs, modification becomes a critical issue in a supportive home environment. Because if older adults do not have access to spaces around the house, due to their physical barriers or social control, they do not feel they can personalize their spaces around the house and tend to avoid adaptation.

The built environment influences the quality of life for older adults. Quality in the environment can slow their physical and mental deterioration. Aging in place is the most strongly held housing preference for older people. Many indicate they like staying in their homes and never moving. However, generally, houses are not designed to meet the needs of older people. The issue is that the aged adapt to their home environment by utilizing different mechanisms: personalization and modification. The need here is to assist older people to remain in their own homes, to help them age in place. The importance of modifying the physical environment increases as older adults continue to expand and respond to their desire to age in place (3, 4). Home modification is crucial because it helps older people age in place, promoting independence by making it easier to perform tasks (5).

Therefore, this research aimed to explore and explain the potential modifications that support the home environment to make older adults feel in control within the cultural context. The study model suggests that older adults will have control over their environment when they feel they have a supportive home environment that allows home modifications. This research is the first of its kind in Jordan. It explored on the meaning of aging and the cultural variations in visioning aging in place within a cultural context. It reflected the importance, benefits, barriers, and priorities for adaptive environments at home. The study examined if modifications in different spaces at home, such as kitchens, bedrooms, living spaces, and reception spaces, are facilitated to provide a supportive environment for older adults to feel in control. This is expected to promote older adults’ independence and well-being and prevent physical accidents and injuries. The hypothesis of the research states that older adults have a supportive environment at home when the overall sense of control is associated with home modification. Expected research outputs include the following: definition of priorities for modifications for spaces to support older adults aging at home.

The decision to select Jordan for this groundbreaking case study, which delves into the meaning of aging and the cultural variations of aging in place, was driven by the unique sociocultural dynamics of the nation that have not been studied comprehensively in this context. This pioneering research serves as a reflection of the significance, benefits, obstacles, and priorities for creating adaptive home environments conducive to aging. The diverse governorates chosen, which include Irbid, Jerash, Ajloun, Mafraq, Zarqa, Salt, and Madaba, represent various distinct cultural, social, and economic spectra within Jordan. This breadth helps to ensure a comprehensive understanding of the experience of aging in different socio-cultural contexts across the nation, enabling a detailed exploration of the topic. Furthermore, there’s value in focusing on such an under-researched area, as it will help fill knowledge gaps and provide meaningful insights into aging in a Middle Eastern context.

## 2 Literature review

This research will extend to linking issues of culture, housing, and community development in relation to older adults.

### 2.1 Culture and aging

Culture and humans are mutual as they shape each other. However, culture is defined by objective and subjective attributes. Objective attributes express materials such as food or clothing; subjective attributes comprise attitudes, values, and beliefs (6). Attitudes, experiences, and behavior in different cultures differ regarding life issues. As individuals age, they face physical, cognitive, and social changes (7–9). Old age can be a successful, active, and productive period (10). Activity theory underlined the importance of continuing activity in old age (11). There are cultural variations in life expectancies, views of older adults, and treatment of older adults. Different cultures have different life expectancies attributed to better medical care, nutrition, and sanitation (7–9). Different cultures hold different cultural values that can affect care from the beginning of life, treatments, and views of independency through aging. As people age around the world, research on cultural attitudes toward aging emerges (12).

Collectivistic cultures embrace positive attitudes towards aging and older adults compared to western cultures because of their value system (13, 14). However, other researchers found contradictory outcomes: more negative attitudes toward aging in Eastern cultures and more positive attitudes toward aging in western culture (12–16). A study showed that two of the top ten countries where older people were perceived as a burden were Arab countries. Where Bahrain is first, Lebanon in third, and Germany in sixth. Meanwhile, of the top ten countries that think older people are treated fairly by the government, three were Arab (14).

### 2.2 Aging in place and control

Usually, older adults fear losing their independence. When older adults move into an institution, they experience a decrease in their freedom and loss of control over their environment. They are forced to leave behind personal items that give meaning to their life and undergo a change in their daily routines and lifestyles (17). In comparison, older adults who live in their own homes have a higher level of control and satisfaction and less loneliness (18–22).

### 2.3 Home modification and aging in place

Harris (23) defined aging in a place where people experience changes over time, but their housing remains unchanged over their lifetime (23). Meanwhile, Lawton and Nahemow (24) defined aging in place as changes to both the person and their surroundings (24). As interaction occurs between people and their surrounding environments, whenever the person’s competence level (functional) changes, the surrounding environment will be dynamically related through modifications. Such environmental continuity will benefit the quality of life and independence (25, 26). People strive to make their delivered housing attributes congruent with their needs through modifications (27, 28). Reasons for people to modify their homes are financial, beautification, and style (29). Older adults who want to remain in their homes generally use measures such as home modifications (30). It potentially poses problems as older adults experience the declines associated with aging (31, 32). To make home environment conducive to maximizing independence, homes should follow universal design principles and adapt existing housing through home modifications. Older adults face impairments while living in homes that lack accommodations to provide support and accessibility. They typically have inaccessible entrances, difficult-to-climb stairs, unsafe bathroom conditions, and narrow hallways and kitchens. Subsequently, they have difficulty moving around the house (33, 34).

Modifications are associated with ownership (35). Home modifications take a different form: removal of throw rugs and provision of assistive gadgets are two examples of a temporary rearrangement of furniture and household equipment (30) to more permanent features in the indoor or immediate outdoor home environment to increase accessibility (i.e., ramp extension, lift installation, and bathroom reconstruction) (36). Home modifications create a supportive home environment. It includes providing adequate space to facilitate care by others and adding supportive features to reduce accidents and falls (3). Home modifications include adapting the home environment to make it easier and safer for older adults to carry out activities such as bathing, cooking, and moving from one space to another. The alterations to the physical structure improve its overall safety and condition to help people age in place (37, 38). Home modifications are more likely to make older adults stay longer at their housing (36, 37, 39). It is positively related to aging in place. Home modifications had positive effects on the quality of life. Home modifications strengthen the personal and social meaning of home for older adults (40). It increases independence and self-confidence by improving activity performance (31, 37).

Among the advantages of the modifications are improved home surroundings’ usability and accessibility (31), enhanced the emotional and social significance of house for older adults (40), reduced dependency in carrying out everyday tasks and decreased caregiving workload (30). Other benefits include (1) accident prevention; (2) aging at home; (3) living in older houses; (4) accommodating lifestyle changes and increasing comfort; (5) engaging in major life activities; (6) cost-effective strategy to reduce health care cost (3, 41, 42). The need for home modification is growing (30). Supporting people to continue living in their homes is less expensive than residential care (25, 39). Many European nations, such as the UK, supported housing initiatives meant to decrease institutional care and encourage home ageing (35). Even when funding was restricted, the expense of home modifications was nonetheless seen as a barrier (43, 44). Home modification barriers are (1) costly and unaffordable to low- and moderate-income individuals; (2) older adults cannot make the changes themselves; (3) lack of awareness of problems of the physical environment and the effectiveness of home modifications; (4) landlords are reluctant to modify the environment to meet the needs of older adults; (5) slow government responses to retrofit subsidized housing or developing regulations for the benefit of older adults (3, 45, 46).

### 2.4 Aging in place and personal characteristics

Providing older adults with options for safe, independent living in the community may improve their quality of life. Aging in place is related to the personal characteristics of older adults: how they perceive, characterize, and address changes in their capacity to live independently and safely in a community. Personal attributes for successful aging in place include resilience, adaptability, independence, and being mentally active and healthy. Aside from knowledge, practical help, money, activity (physical and mental), company (family, friends, neighbours, and pets), transportation and safety, aging in place calls for integrated, responsive, and accessible services (47–49). Older adults with better incomes are more likely to leave their homes but remain in their neighbourhoods to closely preserve their social ties (50).

### 2.5 Recent studies about the adopted one-way ANOVA test

Recent studies using One-Way ANOVA highlight the significance of providing personalized, technologically equipped, and physically adjusted home environments to improve the health, safety, independence, and overall quality of life for older adult individuals. However, more work is needed to understand the myriad of factors that may influence these outcomes, and to develop multi-faceted, universally adaptable design solutions.

The one-way ANOVA (Analysis of Variance) test is a statistical procedure that compares the means of three or more independent groups to determine if there are significant differences amongst them. This technique has been widely utilized in recent years for the investigation of ambient assisted living and the in-home supportive environments for older adults.

Ahn et al. (51) conducted a study employing One-way ANOVA and cross-tabulation analyses to scrutinize significant disparities among different groups in relation to four wellness domains, demographic and housing attributes, as well as the aspiration to age-in-place. The research sought to address an existing gap by exploring perceptions of well-being, which potentially could either stimulate or be the outcome of aging-in-place. The discourse offered insights into how to facilitate successful aging-in-place, underlining the crucial role of the federal government in providing both financial aid and legislation.

Zarghami et al. (52) conducted a study employing One-way ANOVA. This research aims to discover and study the design factors related to improving the quality of life for older adults in residential spaces and choose the best housing option (nursing home, private home, older adult village) for older adults in Iran. The approach was applied through a survey utilizing a questionnaire given to 150 older adults to identify quality of life indicators, and then the significance of these indicators were assessed for various housing options through expert opinion. Thirty-five experts in related fields were selectively consulted for this purpose. The study unearthed five primary factors—educational and encouraging spaces, environmental desirability and comfort, personal tranquility, perception of ownership, and similarity to home—influencing quality of life related to the design of physical spaces. The analysis of these factors suggested that, based on final weighting, the nursing home emerged as the leading housing choice for advancing older adult life quality.

Giuliani et al. (53) conducted a study employing One-way ANOVA. This paper aims to explore the extent to which older adult individuals are open to the use of technology in assisting with day-to-day activities. The focus was on the strategies older adults use to perform their routine tasks at home, to ascertain where technology could be an acceptable aid. Findings indicate that the use of technology is largely contingent on specific problems, whereas personal factors are only significant in unique situations. As people age, there’s a greater tendency to give up, while those with higher education more frequently seek technological solutions.

## 3 Research methods

The core of the study was investigated based on field research. Field research was conducted using two instruments: (a) structured and open-ended interviews; and (b) surveys. Both took place inside the individuals’ houses. The study was carried out over four weeks.

### 3.1 Research design

Field research was a mixed method design as follows:

- Structured and open-ended interviews: interviewing took place at key informants’ homes, among older adults, from different communities in governorates that included: Irbid, Jerash, Ajloun, Mafraq, Zarqa, Salt, and Madaba.
  Sampling technique: convenient sampling was used for the interviews; 35 interviews were conducted.
  Data collection: data was elicited using an open-ended questionnaire.
- Surveys: surveys were conducted by interviewing older adults from a sample of different towns from the above-mentioned governorates.
  Sampling technique: random sampling was used for the surveys. A total sample of 587 from a target of 700 older adults were interviewed at their homes.

Data collection: data was elicited using a structured questionnaire.

### 3.2 Research instrument

The research instruments are as follows:

a) Structured and open-ended interviews: open-ended questions with eight questions. Face-to-face interviews took place at the subject’s home. These questions included:

- What are the services about the village’s infrastructure (was through a reconnaissance survey), typology and housing styles and typology: house heritage and ownership?
- They were asked to describe the social structure at the village and the household level: family members, status, and income
- Inherited values/culture
- They were asked if they feel they have control and how and where
- They were asked about their attachment: where it takes place
- Were there any modifications to the houses allowed?
- They were asked about the objects and tools they keep and what are their values
- They were asked about their favorite/personal space around the house

b) Surveys: a structured questionnaire with 33 questions was given. Surveys took place at the subject’s home.
  Part 1 (Q1-Q18) measuring: confounding variable: Socio-economic characteristics: gender, age, marital status, assigned private rooms, ownership, and length of residence, number of family members, and number of rooms.
  Part 2 (Q19-Q33) measuring: dependent and independent variables as follows:

- Dependent—overall sense of control (Q30): it is measured using two levels: (1) Control 1—Control of Opinion (Average of Q24 + Q25); and (2) Control 2—Control of Activities (Q29).
- Independent 1—space personalization: it is measured using five levels: average of (Q19-Q23)—(P1: Managed Objects + P2: Freedom of Displays+ P3: No Intrusion to Objects + P4: Placement Choice + P5: Fixated (cannot be given).
- Independent 2 (used in this paper)—home modification: it is measured using four levels from (Q27): Average of (Modification of Kitchen + Bedroom +Living Room +Reception Room).
- Independent 3—accessibility to space: it is measured using four levels from (Q26): Average of (Accessibility to Kitchen + Bedroom +Living Room +Reception Room).
- Independent 4—personal space (favorite space around the house): it is defined by favorite space around the house. It is measured using (Q28).

### 3.3 Research setting

**Figure 1:**
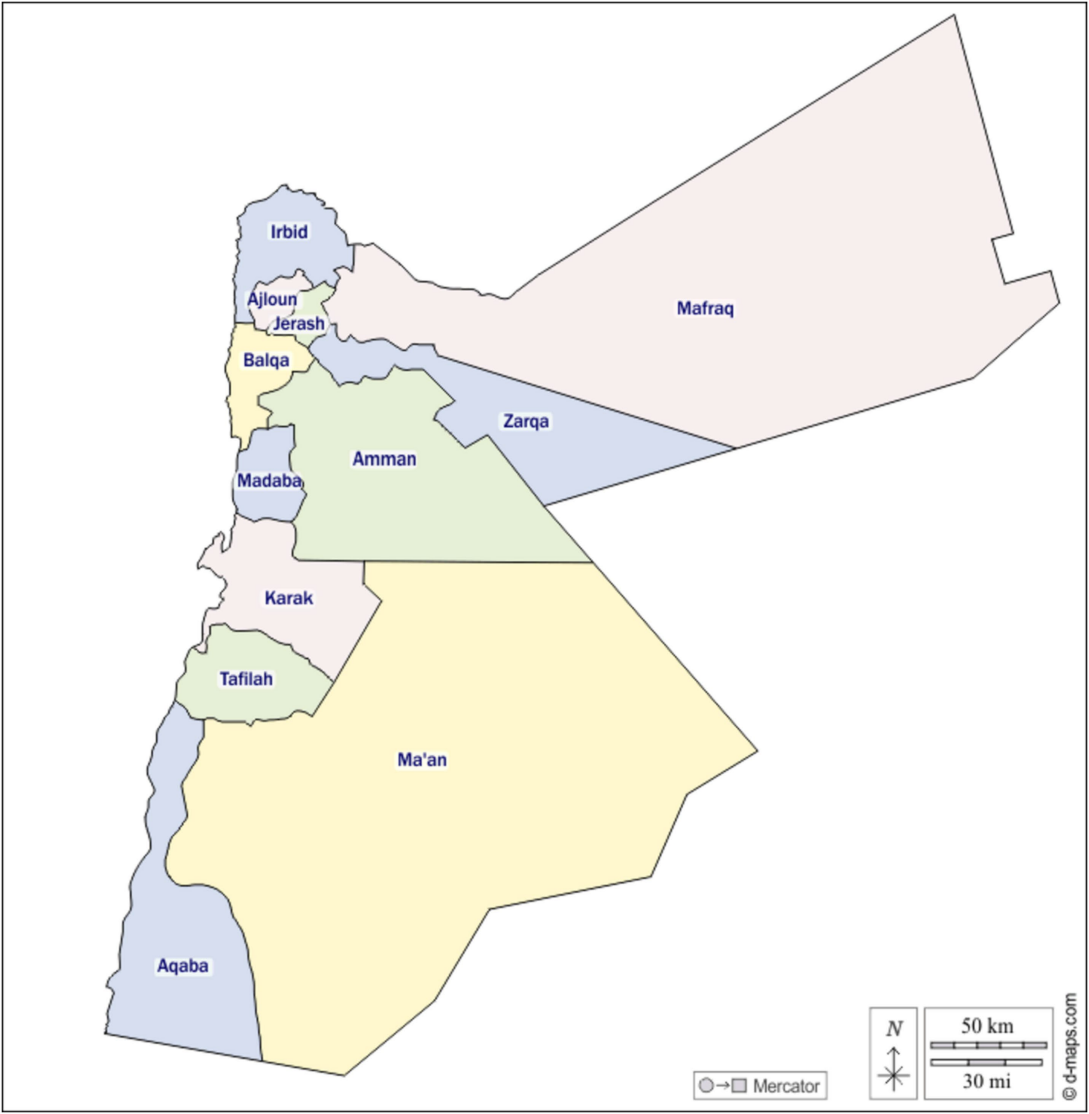
Reprinted with permission from https://d-maps.com/carte.php?num_car=244724&lang=en, © 2007-2024 https://d-maps.com, (Accessed 12.11.2024).

This research was conducted in different governorates of Jordan that, included Irbid, Jerash, Ajloun, Mafraq, Zarqa, Salt, and Madaba, see Figure 1:

1. Irbid: located in the northern part of Jordan, Irbid is the second largest city in the country. It’s known for its educational institutions, including the renowned Yarmouk University. Its geographical location, close to the Syrian border, has also made Irbid a place of refuge for immigrants, contributing to its rich cultural diversity.
2. Jerash: this city, situated in the north of Jordan, is renowned for its well-preserved Roman architecture. Jerash boasts a rich history and is a significant driver of tourism. It’s a mix of cultural heritage and contemporary Jordanian life.
3. Ajloun: nestled in the highlands of north Jordan, Ajloun is famous for its medieval Ajloun Castle and lush forests. The area’s fertile landscapes support a primarily agriculture-based economy.
4. Mafraq: positioned near the borders of Syria, Iraq, and Saudi Arabia, Mafraq has a distinct economy and social structure, largely due to the significant influx of refugees. It’s a hub for industries such as textiles and dairy products.
5. Zarqa: as the industrial center of Jordan, Zarqa houses a significant proportion of the country’s factories and contributes heavily to its economy. It has a vibrant workforce and exhibits an interesting blend of urban and rural styles of living.
6. Salt: salt is a historically rich city located in the Balqa Governorate. Known for its unique Ottoman architectural style, it’s a blend of history, culture, and modern living.
7. Madaba: dubbed as the ‘City of Mosaics,’ Madaba is best known for its Byzantine and Umayyad mosaics, especially the famous 6th century map of Jerusalem and the Holy Land. It has a delicate balance of cultural heritage and modernity.

These diverse locations provide rich grounds for the study, ensuring that a wide array of perspectives across cultural, social, and economic spectra is covered.

### 3.4 Study model

The study models are as follows:

- Older adults have a supportive environment at home when: overall sense of control is associated with home modification (average of 4 levels (dichotomous)—(supportive environment).
- The overall sense of control is correlated with socio-economic characteristics.

### 3.5 Research questions and themes

The following were the study themes and the corresponding questions that were collected through the field interviews:

- General description of the governates and villages: they were asked about their general opinion about the setting.
- Town’s infrastructure: What are the services for the village’s infrastructure (was through a reconnaissance survey)?
- House typology & house heritage and ownership: what are the typology and housing styles and typology: house heritage and ownership?
- Social structure in the village: they were asked to describe the social network of the village.
- The social structure of the household, family members, status, and income: they were asked to describe the social structure at the household level: family members, status, and income.
- Values and culture: inherited values/culture.
- The feel of control and how and where: they were asked if they feel they have control and how and where.
- Attachment: they were asked about their attachment: where it takes place.
- Modifications: were there any modifications to the houses allowed?
- Group conflict: they were asked about group conflict.
- Negative situations and positive emotions: they were asked about negative situations and positive emotions.

### 3.6 Hypotheses of the study

The hypotheses of the study are as follows:

- Hypothesis 1: The older adults have a supportive environment at home when: Overall sense of control is associated with home modification—(supportive environment).
- The sub-hypotheses:
  - Hypothesis 1. a. Home modification is different from Control 1—Control of opinion.
  - Hypothesis 1. b. Home modification is different from Control 2—Control of activities.
- Hypothesis 2: Overall sense of control is correlated with socio-economic characteristics.

### 3.7 Study variables and measures

The variables of the study are as follows:

- Dependent—overall sense of control. It is the ability to have control over the physical and social context of older adults’ lives.
- Independent-home modification: explains how supportive their environment is.
- Confounding variable: socio-economic characteristics.

Variables are measured as follows:

- Dependent—overall sense of control. It is measured using two levels: (1) Control 1—Control of opinion: it is measured using two levels; and (2) control of activities. It is measured using one statement.
- Independent-home modification: it is measured using four levels from: Average of four levels (modification of kitchen, bedroom, living room, and reception room).
- Confounding variable: socio-economic characteristics. It is defined by gender, age, marital status, assigned private rooms, ownership, and length of residence, number of family members, and number of rooms.

### 3.8 Data analysis

- Quantitative analysis includes the following:
  1. A one-way ANOVA analyses were used to analyze significant differences across the groups regarding the mean scores of control of activities with the mean of scores of home modification and its components: modification of kitchen, modification of bedroom, modification of living room, and modification of reception room.
  2. Then a linear regression test was conducted to evaluate the interactive model’s overall sense of control with socio-economic characteristics.
- Qualitative analysis includes the following:
  1. Summary of the interviews
  2. Analysis by extraction of codes: first step: identifying codes from Individual interviews: This involves reading through the interviews transcripts and noting down any specific ideas, concepts, or themes that are discussed). Second step: collapse codes from interviews.
  3. Analysis by extraction of themes: first step: extract themes from Individual interviews. Second step: Collapse themes from Individual interviews.

By analyzing the interview content, we can pinpoint specific codes that highlight the topics covered. These codes act as indicators for the ideas, concepts, or themes explored during the discussions. To thoroughly understand the interview data, further synthesis and analysis were conducted. These codes help organize and categorize the information collected, offering a structured approach to data analysis and addressing particular research questions or objectives. Each code reflects a distinct aspect or theme mentioned in the interview, enabling a more in-depth exploration. From individual interviews, themes were consolidated to identify common overarching themes across all interviews.

## 4 Analysis and discussion

### 4.1 Descriptive analysis

#### 4.1.1 Socio-economic characteristics of the sample

Table 1 displays the descriptive analysis of the sample’s socio-economic characteristics. Almost half the sample from the northern Governates. About 53% of the sample was male subjects. About half of the sample are above 70 years old. One-third of the sample was widowed. About two-thirds of the sample were assigned their private room. Almost all the sample own their own houses. About 60% of the sample lived 10–30 years in the same place, and 40% lived for more than 40 years; the average length of residence is 29 years (M = 29.47). Only about 9% of the sample live alone in their homes; the average family size they live with them is four (M = 4.48). Almost two-thirds of the houses have 1–4 rooms, and one-third above four rooms, with an average room size of four (M = 3.94).

**Table 1:** Descriptive statistics for the sample socio-economic characteristics.

–	Percent	Variance	Skewness	Mean	Std. Deviation
Governorate		3.38	−0.179	3.52	1.84
Irbid	25.6%				
Ajloun	7.2%				
Jerash	14.1%				
Zarqa	11.9%				
Balqa	25.6%				
Madaba	15.7%				
Gender		0.25	0.15	1.47	0.50
Male	53%				
Female	47%				
Age		0.69	−1.09	3.30	0.83
>90 Years	4.4%				
>80–90 Years	10.4%				
>70–80 Years	35.4%				
>60–70 Years	49.7%				
Marital status		0.90	0.67	2.66	0.95
Single	0.9%				
Married	65.4%				
Divorced	0.9%				
Widowed	32.9%				
Assigned private room		0.17	−1.39	1.79	0.41
No	21.5%				
Yes	78.5%				
Ownership		6.12	16.60	2.13	2.48
Charity	6.8%				
Owned	87.6%				
Rented	5.7%				
Length of residence		303.51	0.54	29.47	17.44
≤10	17.30%				
>10–20	20.00%				
>20–30	22.50%				
≥40	40.20%				
Number of family members at home		11.78	0.88	4.48	3.44
None	9.20%				
1–5	56.50%				
>5–10	28.60%				
>10	5.70%				
Number of rooms		3.53	2.06	3.94	1.88
1–4	72.50%				
>4	27.50%				

#### 4.1.2 Descriptive analysis of the dependent and independent variables

The present section shows the distribution for the major study variables. Table 2 displays the descriptive analysis for the dependent and independent variables as follows:

- Dependent—overall sense of control has a mean of M = 4.08 (SD = 1.09) and is defined in terms of (1) Control 1 (Control of opinion) with a mean of M = 4.2 (SD = 1.05) and (2) Control 2 (Control of activities) with a mean of M = 4.32 (SD = 0.90). Control of activities is the highest central tendency among the two attributes. Meanwhile, Control 1 (Control of opinion) has a mean of M = 4.31 (SD = 0.86) and is defined in terms of (1) Control of opinion on general issues has a mean of M = 4.21 (SD = 1.05) and (2) Control of opinion on my issues and the sub-attributes has a mean of M = 4.42 (SD = 0.88). The highest central tendency of which is the modification of the control of opinion on my issues.
- Independent—home modification (explains how to support) (M = 1.59, SD = 0.42) and is defined by modifications of (1) kitchen has a mean of M = 1.51 (SD = 0.50), (2) bedroom has a mean of M = 1.57 (SD = 0.50), (3) living room has a mean of M = 1.64 (SD = 0.48), and (4) reception room has a mean of M = 1.65 (SD = 0.48). The highest central tendency of which is the modification of the reception room.

**Table 2:** Distribution of the dependent and independent variables of the study.

	*N*	Minimum	Maximum	Mean	Std. deviation
Dependent – overall sense of control	587	1.0	6.0	4.08	1.097
Control 1 (control of opinion)	587	1.0	6.0	4.31	0.86
(control of opinion) – consulted on general issues	587	1	6	4.21	1.05
(control of opinion) – Consulted on my issues	587	1	6	4.42	0.88
control 2 (control of activities)	587	1.0	6.0	4.32	0.90
Independent – home modification	587	1.0	2.0	1.59	0.42
Modification of kitchen	587	1.0	2.0	1.51	0.50
Modification of bedroom	587	1.0	2.0	1.57	0.50
Modification of living room	587	1.0	2.0	1.64	0.48
Modification of reception room	587	1.0	2.0	1.65	0.48

### 4.2 Hypotheses testing

#### 4.2.1 Hypothesis 1: home modification is different from the overall sense of control

A One-way ANOVA test was carried out to test the difference in the mean of scores of the overall sense of control with the mean of scores of home modification and its components: modification of kitchen, modification of bedroom, modification of living room, and modification of reception room. Results are presented in Table 3.

**Table 3:** ANOVA of the overall sense of control with home modification and its components.

			Sum of squares	Df	Mean square	F	Sig.
Modification * dependent – overall sense of control	Between groups	(Combined)	11.81	5	2.36	14.71	0.00
		Linearity	10.41	1	10.41	64.83	0.00
		Deviation from linearity	1.40	4	0.35	2.18	0.07
	Within groups		93.33	581	0.16		
	Total		105.14	586			
Modification of kitchen * dependent – overall sense of control	Between groups	(Combined)	12.76	5	2.55	11.08	0.00
		Linearity	10.46	1	10.46	45.40	0.00
		Deviation from linearity	2.30	4	0.58	2.50	0.04
	Within groups		133.89	581	0.23		
	Total		146.65	586			
Modification of bedroom * dependent – overall sense of control	Between groups	(Combined)	11.03	5	2.21	9.66	0.00
		Linearity	9.34	1	9.34	40.89	0.00
		Deviation from linearity	1.69	4	0.42	1.85	0.12
	Within groups		132.78	581	0.23		
	Total		143.82	586			
Modification of living room * dependent – overall sense of control	Between groups	(Combined)	12.03	5	2.41	11.28	0.00
		Linearity	10.04	1	10.04	47.05	0.00
		Deviation from linearity	1.99	4	0.50	2.33	0.06
	Within groups		123.95	581	0.21		
	Total		135.98	586			
Modification of reception room * dependent – overall sense of control	Between groups	(Combined)	12.75	5	2.55	12.21	0.00
		Linearity	11.89	1	11.89	56.97	0.00
		Deviation from linearity	0.86	4	0.21	1.02	0.39
	Within groups		121.26	581	0.21		
	Total		134.00	586			

**Figure 2:**
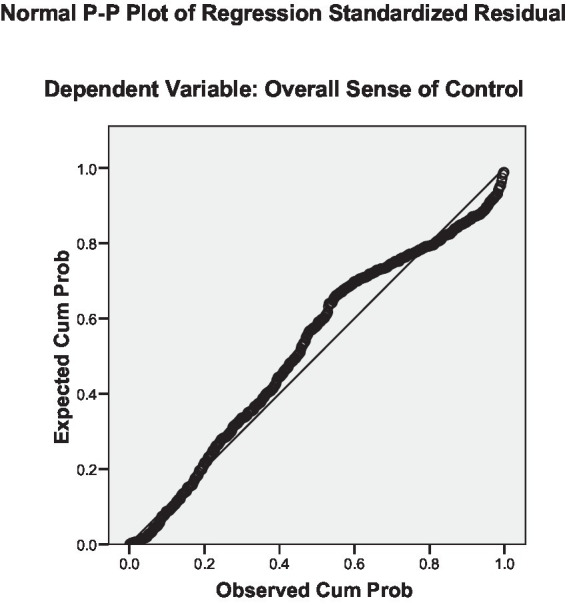
Predicted linear probability plot for the overall sense of control with socio-economic characteristics.

The test indicated a significant effect of home modification and its attributes as follows and in the order of their strength: Home modification [F (5, 586) = 14.71], *p* < 0.00; Reception room modification [F (5, 586) = 12.21], *p* < 0.00; Living room modification [F (5, 586) = 11.28], *p* < 0.00, see Figure 2; Kitchen modification [F (5, 586) = 11.08], *p* < 0.00; and bedroom modification [F (5, 586) = 9.66], *p* < 0.00. The reception room is considered a semi-public space in the house, and it is kind of the center of events in the house, so being able to modify this space makes older adults feel in control and influence in daily life. On the other hand, bedrooms are private cocoons, modifying them would not necessarily make them feel the most influential and in control.

#### 4.2.2 Hypo 1. a. home modification differs from control 1—control of opinion

A One-way ANOVA test was carried out to test the difference in the mean of scores of Control 1—Control of opinion with the mean of scores of home modification and its components: modification of kitchen, modification of bedroom, modification of living room, and modification of reception room. Results are presented in Table 4.

**Table 4:** ANOVA of control 1 – control of opinion with home modification and its components.

			Sum of squares	Df	Mean square	F	Sig.
Modification * control 1	Between groups	(Combined)	7.72	10	0.77	4.56	0.00
		Linearity	5.05	1	5.05	29.84	0.00
		Deviation from linearity	2.67	9	0.30	1.75	0.07
	Within groups		97.42	576	0.17		
	Total		105.14	586			
Modification of kitchen * control 1	Between groups	(Combined)	9.09	10	0.91	3.81	0.00
		Linearity	5.32	1	5.32	22.27	0.00
		Deviation from linearity	3.77	9	0.42	1.75	0.07
	Within groups		137.56	576	0.24		
	Total		146.65	586			
Modification of bedroom * control 1	Between groups	(Combined)	6.71	10	0.67	2.82	0.00
		Linearity	4.75	1	4.75	19.95	0.00
		Deviation from linearity	1.96	9	0.22	0.91	0.51
	Within groups		137.11	576	0.24		
	Total		143.82	586			
Modification of living room * Control 1	Between groups	(Combined)	9.85	10	0.99	4.50	0.00
		Linearity	6.71	1	6.71	30.64	0.00
		Deviation from linearity	3.14	9	0.35	1.59	0.11
	Within groups		126.14	576	0.22		
	Total		135.98	586			
Modification of reception room * Control 1	Between groups	(Combined)	7.27	10	0.73	3.30	0.00
		Linearity	3.65	1	3.65	16.59	0.00
		Deviation from linearity	3.62	9	0.40	1.83	0.06
	Within groups		126.74	576	0.22		
	Total		134.00	586			

The test indicated a significant effect of home modification and its attributes as follows and in the order of their strength: Home modification [F (10, 586) = 4.56], *p* < 0.00; Living room modification [F (10, 586) = 4.49], *p* < 0.00; Kitchen modification [F (10, 586) = 3.81], *p* < 0.00; Reception room modification [F (10, 586) = 3.30], *p* < 0.00; and bedroom modification [F (10, 586) = 2.82], *p* < 0.00. Bedrooms are private cocoons; modifying them would not necessarily make them feel the most influential and in control.

#### 4.2.3 Hypo 1. b. home modification is different from control 2 – control of activities

The difference in the mean scores of control 2 was examined using a One-way ANOVA test. – Control of activities with the mean of scores of home modification and its components: modification of kitchen, modification of bedroom, modification of living room, and modification of reception room. Results are presented in Table 5.

**Table 5:** ANOVA of control 2—control of activities with home modification and its components.

			Sum of Squares	Df	Mean Square	F	Sig.
Modification *– control 2	Between groups	(Combined)	13.48	5	2.70	17.09	0.00
		Linearity	9.93	1	9.93	62.94	0.00
		Deviation from linearity	3.55	4	0.89	5.62	0.00
	Within groups		91.66	581	0.16		
	Total		105.14	586			
Modification of kitchen * control 2	Between groups	(Combined)	14.77	5	2.95	13.01	0.00
		Linearity	11.62	1	11.62	51.21	0.00
		Deviation from linearity	3.14	4	0.79	3.46	0.01
	Within groups		131.89	581	0.23		
	Total		146.65	586			
Modification of bedroom * control 2	Between Groups	(Combined)	12.95	5	2.59	11.5	0.00
		Linearity	9.90	1	9.90	43.93	0.00
		Deviation from linearity	3.05	4	0.76	3.39	0.01
	Within groups		130.87	581	0.23		
	Total		143.82	586			
Modification of living room * control 2	Between groups	(Combined)	14.05	5	2.81	13.39	0.00
		Linearity	9.88	1	9.88	47.09	0.00
		Deviation from linearity	4.17	4	1.04	4.97	0.00
	Within groups		121.93	581	0.21		
	Total		135.98	586			
Modification of reception room * control 2	Between groups	(Combined)	13.03	5	2.61	12.51	0.00
		Linearity	8.44	1	8.44	40.54	0.00
		Deviation from linearity	4.58	4	1.15	5.50	0.00
	Within groups		120.98	581	0.21		
	Total		134.00	586			

The test indicated a significant effect of home modification and its attributes as follows and in the order of their strength: home modification [*F* (10, 586) = 17.09], *p* < 0.00; living room modification [F (10, 586) = 13.39], *p* < 0.00; kitchen modification [F (10, 586) = 13.01], *p* < 0.00; reception room modification [F (10, 586) = 12.51], *p* < 0.00; and bedroom modification [F (10, 586) = 11.49], *p* < 0.00. Bedrooms are private cocoons; modifying them would not necessarily make them feel the most influential and in control.

#### 4.2.4 Hypothesis 2: overall sense of control is correlated with socio-economic characteristics

A linear regression test was conducted to evaluate the interactive model’s overall sense of control with socio-economic characteristics. For this model, the interactive relationship is reported as significant in Table 6: [*F* (8, 586) = 13.31], *p* < 0.00. This suggests that socio-economic characteristics are associated differently with older adults’ sense of control.

**Table 6:** Linear regression model summary – overall sense of control with socio-economic characteristics.

Model		Sum of squares	df	Mean square	F	Sig.
1	Regression	109.73	8	13.72	13.31	0.00
	Residual	595.51	578	1.03		
	Total	705.24	586			

Results of the significant effect of individual mean association for each component in the model are shown in Table 7. The strength of the individual contribution to the model are listed in order as follows: age [t = 4.59, *p* = 0.00], Number of family members at home [t = −4.59, *p* = 0.00], Assigned private room [t = 3.49, *p* = 0.00], Number of rooms [t = 3.33, *p* = 0.00], Gender [t = −3.23, *p* = 0.00], and marital status [t = −2.87, *p* = 0.00]. The following showed a negative direction: Number of family members at home, gender, and marital status.

**Table 7:** *T*-test for socio-economic characteristics in the linear logistic regression model.

Model		Unstandardized coefficients		Standardized coefficients	*t*	Sig.
		B	Std. Error	Beta	B	Std. Error
1	(Constant)	3.28	0.35		9.28	0.00
	Gender	−0.30	0.09	−0.14	−3.23	0.00
	Age	0.27	0.06	0.20	4.59	0.00
	Marital status	−0.14	0.05	−0.13	−2.87	0.00
	Assigned private room	0.37	0.12	0.14	3.49	0.00
	Ownership	−0.02	0.02	−0.04	−0.96	0.34
	Length of residence	0.00	0.00	0.03	0.82	0.41
	Number of family Members at home	−0.06	0.01	−0.19	−4.59	0.00
	Number of rooms	0.08	0.03	0.141	3.33	0.00

Regression residual shows normal fitness between the predicted and the estimated data for the overall sense of control relationship with socio-economic characteristics with M = 2.69, SD 0.99. It shows high fitness between the two models and a normal distribution. Meanwhile, Figure 2 displays the predicted linear probability plot for the overall sense of control with socio-economic characteristics. The relationship demonstrates linearity.

### 4.3 Qualitative outcomes—interviews themes

The interview breakdown elucidated the following central themes into detail:

**Theme 1: General description of the governates and villages:** Clustered villages with a physical setting in a rural environment; hierarchical from public to private; demarked with territorial markers and fences; villages have sceneries and views from all directions; farming and livestock are the lifestyles; some villages have apartment buildings, especially when commercial is present; schools are there; and fences exist in different heights depending on the village location.

**Theme 2: Town’s infrastructure is as follows:** Random street layout with random housing typology; service includes a mosque, health centre, schools, and neighbourhood parks; local people gave land to the local governorates to provide services; some streets are not paved; not entertainment services for the youth; some area has no electricity; some villages have no playing grounds or local parks; some village has physical open gates giving the feel of a gated community; local market; and transportation.

**Theme 3: House typology & house heritage and ownership as follows:** Some houses are old construction from basalt and stone and others from concrete or concrete blocks; traditional houses have cross vaults and kuwrar for food storage; house typology is almost the same all over the interviewed villages; house have wells for drinking water collected from rain; fences are low and symbolic, and some villages have apartments on top of commercial strips that serve the village as a local market.

**Theme 4: Social structure of the village:** kinship and social and communal, socializing and visiting, perception of the past is more social more sacred, bonding lifestyle in the villages; education is only high school; they are holding periodical meetings for older adults to discuss community issues; good neighbouring relationships – they drink coffee together at one of the houses or the local shop, and they play chess; the village became huge, and people became isolated socially; and identity changed in some villages because of working and studying outside the village or outside Jordan.

**Theme 5: Social structure of the household, family members, status, and income:** oldest older adults in the village, 90 years old; lived as an extended family; income is ok: farming, livestock, military. Few have bachelor’s degree – middle income; inherited house and owned; land owned; trees olives; basic furniture; and health is ok.

**Theme 6: Values and culture:** older adult care; older adult respect; passing control to older adults; bonding; socializing; communal; generosity; welcoming; friendly; religious; and self-sufficient.

**Theme 7: Feel of control and how and where:** they are consulted on many issues; they control all family members, and they are cooperative; they have control over the house and over everything and all objects; males have more control than females; those who live alone have more control and if they share with their sons they feel in less control; control makes them feel alive and happy; private families have older adults in control more than others; the less family member is around the more control they feel they have; if they have an assigned private space and room, they feel they have more control than those who do not; control decreases with age and health; 60–70 have so much control; and 70–80 80% control relative control and 20% have full control. There were three types of control: physical and social on everything, social and opinion only; some did not feel they needed to control anything, and some had no control.

**Theme 8: Attachment:** to the village and the house and the family and the community; attached to their tools; attached to land and farming and agriculture not to tools; gender difference reflects different attachment, females are more attached; some attached to these objects because of their symbolic and emotional values, especially if they are handmade and how it has a cultural meaning; attachment to belonging comes from memories; and health, income, affect their feel of control.

**Theme 9: Modifications:** they often upgraded the house because of family size.

**Theme 10: Group conflict:** age group 20–30 years old.

**Theme 11: Negative situations:** some older adults were lonely and isolated, poor, no care, and ignored; reasons include their children being busy; some houses are not suitable for older adults, especially the bathroom, windows, and doors are not isolated; and poor construction of the walls that is cracked.

**Theme 12: Positive emotions:** older adults needs support and care; their soul is beautiful; tradition guarantees keen life for the older adults as a culture.

The themes can be consolidated into two overarching categories related to overall sense of control and home modification as follows:

A) **Themes related to sense of control**

This theme encompasses the various aspects of control experienced by individuals in the community, highlighting the interactions between social structures, cultural values, and individual agency. Key sub-themes include:

1. Social and familial control: the influence of family dynamics and social structures on individual control, with specific emphasis on the roles of older adults and the gender differences in control. Males often have more control, while those living alone or in smaller family units report a greater sense of autonomy. Periodic community meetings foster a sense of collective decision-making.
2. Control within the home: control over living spaces affects individuals’ well-being, with allocated personal space enhancing feelings of autonomy. The relationship between health and age significantly influences perceived control; older adults may feel less empowered as they age.
3. Community engagement: residents express control through participation in decision-making, fostering connections within the village. The social fabric also plays a role in maintaining a sense of belonging and community identity, despite trends of isolation.

B) **Theme related to home modification**

This theme reflects the adaptations made to living spaces and the cultural significance of home. It includes:

1. Physical structure and modifications: homes are often modified to accommodate family size and evolving needs. The typology of houses—ranging from traditional stone structures to more modern constructions—impacts both functionality and comfort. Challenges faced by older adults regarding suitability of homes highlight the need for improvements in infrastructure, particularly for accessibility.
2. Emotional and symbolic attachments: there is a deep attachment to homes, land, and community, which is shaped by personal and collective memories. Modifications often reflect a desire for maintaining connections to cultural heritage and family legacies, with significance placed on objects and spaces that carry emotional weight.

These condensed themes illustrate the interplay between individual control and home modifications, influencing the quality of life in rural communities.

### 4.4 Comparative outcomes

Looking at the analysis of the hypotheses, it appears the data from both the quantitative statistics and the qualitative interviews complement each other and provide comprehensive insights into older adults’ sense of control based on home modifications. This multidimensional understanding helps enhance the originality of the research, as it interrelates different aspects: the physical environment, socio-economic attributes, and deeply rooted cultural factors affecting the perception of control among older adults.

The data collected through this research highlights the relationship between home modification, the overall sense of control and socio-economic characteristics of older adults. However, to ensure a robust analysis, the information gained through both qualitative interviews and quantitative questionnaires should be integrated.

Comparing the interview data to the results of the hypothesis testing, a clear pattern emerges. Older adults feel a stronger sense of control when they are able to modify their living spaces, especially the semi-public spaces such as the reception and living rooms. This is reflected in the qualitative themes, which highlight a strong attachment to the home and the need for control over surroundings and objects. The findings indicate that modifications provide a sense of instrumental and psychological control, influencing the quality of life of older adults.

The ANOVA test results identified a significant interactive effect of home modification components and different layers of control, whether it is overall control, control of opinion (Hypo 1.a), or control of activities (Hypo 1.b). This supports the qualitative finding that different levels of control are perceived by older adults, and few have no or limited control.

Relating to Hypothesis 2, it’s observed that socioeconomic characteristics significantly correlate with the older adults’ sense of control. This was supported by qualitative interviews where participants expressed the effect of factors such as age, gender, and number of family members at home on their sense of control. A deeper exploration of these characteristics could yield richer insights into the societal and cultural patterns that influence the sense of control.

From the qualitative interviews, it’s also apparent that the villages’ social fabric and infrastructure, family status, income, values, and culture significantly influence older adults’ feelings of control. These variables, while not directly tested in the quantitative data, provide crucial context and depth to our understanding.

Note that the interview data and questionnaire results also reflect the potentially negative implications of an older adult’s lack of control. The regression residuals suggest a high fit between the predicted and estimated relationships, but they hint towards certain socio-economic characteristics leading to negative situations like isolation and poor care.

To delve deeper into these relationships, comparing the quantitative and qualitative findings reveals several noteworthy points:

1. **Home modifications & sense of control**: both the ANOVA results and the qualitative themes underscore the significant association between different kinds of home modifications and the sense of control among older adults. The modifications, especially in communal areas like the living room and reception area, seem to empower older adults as they can influence their daily living spaces, thereby feeling more in control.
2. **The interplay of privacy and control**: the difference in the perceived sense of control depending on whether modifications are done in public areas versus private spaces (like bedrooms) is evident in both the results obtained from the ANOVA and reflected in themes from the interviews. The bedrooms, being personal sanctuaries, do not necessarily enhance older adults’ sense of control when modified, while common areas apparently do.
3. **Correlation with socio-economic characteristics**: the quantitative results suggest a significant correlation between socio-economic factors (like age, number of family members at home) and the overall sense of control. This is echoed in the qualitative analysis where the older adults’ feelings of control are also influenced by family structures, the number of family members at home, and issues like familial respect and control. This highlights how socio-economic conditions can tangibly impact older adults’ emotional wellbeing and their perceived control and influence.
4. **Sense of control in different contexts**: the qualitative findings highlight numerous factors that influence older adult individuals feelings of control, such as influencing family members, control over personal objects, health status, or even having private spaces in the house. These are unique insights that supplement the quantitative analysis, demonstrating the importance of context and personal factors when relating to older adults’ sense of control.
5. **Town infrastructure & heritage**: while not directly arising from the hypotheses, themes about the town infrastructure, house typology, and heritage provide relevant contexts that might be influencing the feeling of control. The living conditions provided by the town’s infrastructure, the traditional or modern structure of the homes, and historical attachments can all be potential factors influencing the older adults’ feelings of control.
6. **Cultural aspects**: this research shines light on community values and culture which indeed shapes the older adults’ sense of control. Themes reveal a culture of respect for older adults, and importance of communal social structures, which might influence their perceived control more than the physical modifications of the home. These cultural insights supplement and enrich the quantitative findings, bringing in unique local perspectives.

In summary, an interlinked understanding of both the quantitative and qualitative findings offers a much richer perspective on the interplay between home modifications, socio-economic characteristics, and cultural elements in influencing older adults’ sense of control. This integrated approach enhances the originality of the study and provides a deeper understanding of the topics at hand. In conclusion, comparing and integrating these two data sources provides a clearer, more comprehensive understanding of the factors influencing older adults’ sense of control related to home modifications.

Future studies could further explore the nuanced influences of specific socio-economic characteristics and environmental factors on feelings of control.

### 4.5 Unintended outcomes and barriers

The study highlights the following:

1. Home modifications: significant association between various modifications in communal living spaces (like living rooms and reception areas) and the increased sense of control experienced by older adults. Modifications here help older adults feel empowered by providing them influence over their living environment.
2. Town infrastructure & heritage: while not directly highlighted in the hypotheses, modifications related to the town’s infrastructure, house typology, and heritage are seen to potentially affect older adults’ sense of control by providing contexts for modifications.

Potential barriers for home sense of control and modifications include:

- Socio-economic characteristics: age, gender, family structures, and quantity of family members can act as barriers, affecting the sense of control among older adults. Certain socio-economic conditions might lead to negative outcomes such as isolation and poor care.
- Cultural aspects: cultural values and societal structures could pose barriers where older adults may have limited perceived control despite physical home modifications, if cultural values do not support older adult empowerment or personal independence within the community or family.
- Privacy in modifications: modifications in private spaces such as bedrooms do not necessarily enhance older adults’ sense of control, unlike those in communal/shared spaces.

On the other hand, unintended Consequences of home modifications include:

- Lack of control and negative implications: the regression analysis suggests that certain socio-economic characteristics can lead to negative situations like isolation and deficient care for older adults, despite modifications intended to empower them.
- The interplay of privacy and control: while modifications in public areas enhance the sense of control, changes in private spaces may not yield the same result, potentially leading to less satisfaction or empowerment than expected.

These insights underline the complexity of enhancing control among older adults through home modifications and highlight the need for a thoughtful approach considering socio-economic and cultural factors to avoid undesired effects.

## 5 Conclusion

Almost half the sample was from the northern governates. Half the sample were male subjects. About half of the sample was above 70 years old, one-third were widowed, and about two-thirds had their private room. Almost all the sample own their own houses. About 60% of the sample lived 10–30 years in the same place, and 40% for more than 40 years. The average length of residence is 29 years. Only about 9% of the sample live alone in their homes. The average number of family members they live with is four. Almost two-thirds of the houses have 1–4 rooms, and one-third have more than four. The average house size is four rooms. The older adults enjoyed social attachment and controlled more than physical attachment and control.

### 5.1 Conclusive relationships

#### 5.1.1 Supportive environment at home

1. Overall sense of control is associated with home modification (supportive environment):
2. Home modification is different with overall sense of control: Overall sense of control with home modification and its components: modification of kitchen, modification of bedroom, modification of living room, and modification of reception room indicated a significant effect in the order of their strength: home modification; reception room modification; living room modification; kitchen modification; and bedroom modification.
3. Home Modification is different with Control 1—Control of opinion: Control 1—Control of opinion with home modification and its components: modification of kitchen, modification of bedroom, modification of living room, and modification of reception room indicated a significant effect in the order of their strength: home modification; living room modification; kitchen modification; reception room modification; and bedroom modification.
4. Home Modification is different from Control 2—Control of activities: Control 2—Control of activities with home modification and its components: modification of kitchen, modification of bedroom, modification of living room, and modification of reception room indicated a significant effect in the order of their strength: home modification; living room modification; kitchen modification; reception room modification; and bedroom modification.

#### 5.1.2 Social impact on adaptive environment at home

Overall sense of control is correlated with socio-economic characteristics: overall sense of control with socio-economic characteristics are associated as follows: age, number of family members at home, assigned private room, number of rooms, and marital status. A negative direction: number of family members at home, gender, and marital status.

#### 5.1.3 Comparative conclusions

Drawing from both the qualitative and quantitative aspects of the data shared, I can provide the following comparative conclusions:

1. **Influence of home modifications**: there is a clear correlation between home modifications and an enhanced sense of control experienced by older adults. Modifications in shared spaces were notably impactful, offering a strong sense of control and influence over their daily lives across both qualitative and quantitative data.
2. **Public vs private spaces**: the distinction between shared and private areas was prominent. While communal spaces like living rooms and reception rooms enhanced the sense of control when modified, changes in private areas, specifically bedrooms, did not seem to influence the older adults’ perceived control as per both the hypotheses tested and the qualitative narratives.
3. **Socio-economic interplay**: socio-economic factors exert a substantial influence on the older adults’ sense of control. The statistical analysis pointed towards key contributors such as age and the number of family members at home. This finding was supported by the interview data, where familial structures and respect, along with other socio-economic conditions, significantly influenced their sense of control.
4. **Control in context**: the qualitative input expanded upon the statistical data by highlighting the influence of context and personal factors on older adults’ sense of control. Elements such as social opinion, personal health status, and degree of privacy showed importance alongside physical factors like home modifications.
5. **Importance of infrastructure and heritage**: despite not being a focal point of the hypotheses, the impact of the town’s infrastructure, house typology, and cultural heritage on the older adults’ sense of control came through in the interview data. This is a context that indirectly supports the results from the statistical analysis.
6. **Cultural sensitivities**: the study uncovered a layer of complexity presented by cultural factors. While the equations and statistics did not explicate this, the qualitative themes unveiled the culture of respect for older adults and communal social structures that deeply influence their perceived control, sometimes even surpassing physical factors.

Overall, the conclusions derived through data triangulation – combining statistical or quantitative data with qualitative data – have enriched the comprehensiveness of this study. By understanding the role of home modifications, socio-economic characteristics, and cultural dimensions, we have a much deeper insight into older adults’ perceived control within their living environments. This opens up the possibility for further research to deepen these findings or to apply them in practical situations aiming to improve the quality of life for our older adults population.

#### 5.1.4 Future models of investigation

Based on the qualitative outcomes, future research frameworks are suggested as follows:

Based on the qualitative outcomes and themes identified, future frameworks for studies in this context can be structured to address specific needs and areas of interest. Below are some suggested frameworks:

1 Socio-cultural dynamics and identity preservation framework:

- **Objective:** To explore and preserve socio-cultural identity in the face of modernization and external influences.
- **Components:**
- Examine the role of cultural heritage in villages’ identity and how modernization affects it; propose strategies for preservation and adaptation.
- Study the impact of migration on community dynamics and attachment, considering emotional and symbolic connections.
- Develop initiatives to promote cultural exchange between villages and external entities, ensuring cultural continuity.

2 Home adaptation and sustainable living framework:

- **Objective:** To ensure homes are adaptable to changing needs and sustainable for future generations.
- **Components:**
- Evaluate the structural integrity and functionality of existing homes, focusing on traditional versus modern building techniques.
- Promote sustainable practices in home modifications, such as rainwater harvesting and energy-efficient designs, that align with local lifestyles.
- Encourage community-led home modification initiatives that allow sharing of skills and resources, fostering collective responsibility and empowerment.

3 Empowerment and autonomy framework for older adults:

- **Objective:** To enhance the autonomy and quality of life for older adults in rural communities.
- **Components:**
- Establish support systems for older adults, integrating health care, social activities, and community involvement.
- Research the effectiveness of traditional structures in providing control and propose adaptations to strengthen social support networks.
- Develop platforms for knowledge transfer where older adults can share their wisdom and experiences, reinforcing their roles within families and the community.

4 Conflict resolution and cohesion building framework:

- **Objective:** To address and mitigate intergenerational and social conflicts within the community.
- **Components:**
- Implement conflict resolution workshops focusing on intergenerational understanding and cooperation.
- Promote activities that encourage social cohesion, such as group projects or community events that unite different demographic groups.
- Monitor changes in community dynamics regularly to address any emerging issues proactively.
- These frameworks provide a focused approach to understanding and improving rural community life, addressing both immediate and long-term challenges by integrating social, cultural, and structural elements.

### 5.2 Recommendations and implications

As discussed in the research, aging means growing without having to move to another place. Providing a supportive environment at home is the key to carrying out tasks and daily activities for older adults. Promoting well-managed, regulated home care services is essential since doing so would enable older people to receive care while still living with their families and communities. Therefore, caregivers can be provided at home to maintain health and well-being. In addition, physical provisions should be for the convenient physical layout to house different activities for older adults without facing any obstacles. This can only occur by boosting awareness about the existing problems in the physical environment and the benefits of modification and barriers.

Recommended changes at home to enhance safety, health, security, and self-maintenance should take place at the following paces:

- Bathrooms: Install hinges to doors, widen doors, adjustable furniture, grab bars, handles, round counters, and nonskid tiles.
- Kitchens: round counters, switches, door handles, widen doors, adjustable furniture.
- Bedrooms, living rooms, and reception rooms: widen doors, widen pathways, ventilation, heating and cooling, nonskid floors.
- Halls, doors, entrances and exits clear pathways, door handles, nonskid floors, widen doors, lighting.
- Enhance energy efficiency and air quality and view as they affect health.

To provide control for those who age at place, planning is needed, and the following should be developed:

- Modifications: home modifications and adaptation to changes in physical and psychological needs: (1) Places to modify: Bathrooms, kitchens, bedrooms, living rooms, doors, halls, entrances, and exits. (2) Details to modify confining living space to one floor, installing electric chairs and elevators if needed, modifying bathroom and kitchen detailing and heights of tops and storage areas.

#### 5.2.1 Modification guidelines

To facilitate a supportive and safe home environment for older adults that promotes a sense of control and independence, here are detailed design guidelines for modifications across various areas of the home. The intent is to ensure such modifications effectively address physical and psychological needs, improving the overall quality of life for aging individuals.

##### 5.2.1.1 General principles

User-centered design: involve older adults residents in the modification process to ensure their needs and preferences are reflected in the design.

Accessibility standards: follow local accessibility regulations like ADA (Americans with Disabilities Act) to ensure proper compliance.

1 Bathroom modifications

- Doorways:
- Widen bathroom doors to at least 32 inches for easy wheelchair access.
- Install doors that swing outward or pocket doors to save space.
- Flooring:
- Use nonskid tiles or vinyl flooring to reduce slip risk.
- Ensure floors are level transitions between areas (remove threshold).
- Grab bars and handles:
- Install grab bars beside the toilet, in the shower, and near the tub.
- Use lever-style door handles for ease of use.
- Adjustable fixtures:
- Consider installing adjustable height vanities for flexibility.
- Use handheld showerheads to provide ease of use for bathing.

2 Kitchen modifications

- Layouts:
- Design a U- or L-shaped kitchen layout to minimize distance and facilitate movement.
- Ensure standing areas have clear pathways.
- Surfaces:
- Use rounded countertops to prevent injury from sharp corners.
- Install adjustable countertops and shelves to reach items easily.
- Appliances:
- Select appliances with touch controls or simple dials.
- Install appliances at waist height to minimize bending and reaching.

3 Living areas (bedrooms, living rooms, reception rooms)

- Doorways and pathways:
- Widen doorways and maintain a minimum 36-inch clear pathway to support mobility aids.
- Remove rugs and other tripping hazards.
- Ventilation and lighting:
- Ensure adequate ventilation, with windows that can be opened easily.
- Install bright, energy-efficient lighting with dimmer switches.
- Flooring:
- Use nonskid flooring throughout all living spaces for safety.
- Consider carpets with low pile for trips and falls prevention.
- Halls, entrances, and exits
- Pathway Clearance:
- Keep hallways clear of furniture and obstacles to support mobility.
- Maintain wide pathways and ensure door handles are accessible.
- Lighting:
- Install motion-sensor lights for safety when entering hallways or exits.
- Use contrasting colors for walls and floors to assist people with vision impairments.
- Energy efficiency and air quality enhancements
- Windows and Insulation:
- Upgrade windows for better thermal conductance (double-glazing).
- Ensure sufficient insulation to enhance heating/cooling efficiency.
- Air quality:
- Install air purifiers and ensure proper ventilation systems are in place to maintain air quality.
- Use non-toxic paints and building materials to minimize potential health risks.

4 Planning for future modifications

- Single-floor living:
- If possible, design homes to be suitable for single-floor living to reduce stairs’ challenges.
- Mobility aids:
- Prepare for the installation of stairlifts or elevators in multi-story homes.
- Assess for grab bars or lifts in all critical areas (e.g., bathrooms, kitchens).

Implementing these guidelines creates a well-adapted living space that enhances safety, supports health, and promotes self-maintenance among older adults. By empowering them with modifications that respond to their evolving needs, caregivers and family members can foster an environment that nurtures independence and dignity. Continuous evaluations and adjustments should be made to ensure the modifications remain effective as needs change over time.

### 5.3 Implications on housing sector for older adults

To implement the study outcomes, changes will have to be made in various areas for housing for older adults around Jordan whenever needed: home design and neighbourhood design. This infusion in the housing market provide better urban policies for urban design and urban planning based on cultural heritage and values related to older adults. As a learning lesson, the following stakeholders can be involved and as follows:

1. Decision makers: an integrated strategy for creating community-based housing for older adults’ accessibility to community-based housing facilities offer reasonably priced services and will improve older people’s ability to age in place.
2. The ministry of health and social services: it also has an important role to play regarding infusing and developing housing regulations for older adults. Infuse building codes related to older adults accessibility, especially in universal design.
3. Municipality: at the municipality’s level, new regulations may allow mixed land use where houses and shops are provided in the same area. Safe communities and adaptable physical environments must be encouraged to encourage older persons to walk, use public transit, and participate in social activities.
4. Architects: architects and developers must create new housing models that accommodate older people’s need for privacy, social interaction, and control.
5. Non-governmental organizations and not-for-profit societies: they have the potential to respond to older adults needs regarding home maintenance.

## Data Availability

The original contributions presented in the study are included in the article/supplementary material, further inquiries can be directed to the corresponding author.
